# Classifying Parkinson’s Disease Patients With Syntactic and Socio-emotional Verbal Measures

**DOI:** 10.3389/fnagi.2020.586233

**Published:** 2020-11-23

**Authors:** Sandra Baez, Eduar Herrera, Catalina Trujillo, Juan F. Cardona, Jesus A. Diazgranados, Mariana Pino, Hernando Santamaría-García, Agustín Ibáñez, Adolfo M. García

**Affiliations:** ^1^Department of Psychology, Universidad de los Andes, Bogotá, Colombia; ^2^Departamento de Estudios Psicológicos, Universidad Icesi, Cali, Colombia; ^3^Instituto de Psicología, Universidad del Valle, Cali, Colombia; ^4^Centro Médico de Atención Neurológica “Neurólogos de Occidente”, Cali, Colombia; ^5^Department of Psychology, Universidad Autónoma del Caribe, Barranquilla, Colombia; ^6^Centro de Memoria y Cognición, Intellectus-Hospital Universitario San Ignacio, Bogotá, Colombia; ^7^Department of Psychiatry-Physiology and Ph.D. Program in Neuroscience, Pontificia Universidad Javeriana, Bogotá, Colombia; ^8^Universidad de San Andrés, Buenos Aires, Argentina; ^9^National Scientific and Technical Research Council (CONICET), Buenos Aires, Argentina; ^10^Center for Social and Cognitive Neuroscience (CSCN), School of Psychology, Universidad Adolfo Ibáñez, Santiago de Chile, Chile; ^11^Global Brain Health Institute, University of California, San Francisco, San Francisco, CA, United States; ^12^Faculty of Education, National University of Cuyo (UNCuyo), Mendoza, Argentina

**Keywords:** Parkinson’s disease, sentential processing, multidimensional assessment, syntactic processing, social emotions

## Abstract

Frontostriatal disorders, such as Parkinson’s disease (PD), are characterized by progressive disruption of cortico-subcortical dopaminergic loops involved in diverse higher-order domains, including language. Indeed, syntactic and emotional language tasks have emerged as potential biomarkers of frontostriatal disturbances. However, relevant studies and models have typically considered these linguistic dimensions in isolation, overlooking the potential advantages of targeting multidimensional markers. Here, we examined whether patient classification can be improved through the *joint assessment* of both dimensions using sentential stimuli. We evaluated 31 early PD patients and 24 healthy controls *via* two syntactic measures (functional-role assignment, parsing of long-distance dependencies) and a verbal task tapping social emotions (envy, *Schadenfreude*) and compared their classification accuracy when analyzed in isolation and in combination. Complementarily, we replicated our approach to discriminate between patients on and off medication. Results showed that specific measures of each dimension were selectively impaired in PD. In particular, joint analysis of outcomes in functional-role assignment and *Schadenfreude* improved the classification accuracy of patients and controls, irrespective of their overall cognitive and affective state. These results suggest that multidimensional linguistic assessments may better capture the complexity and multi-functional impact of frontostriatal disruptions, highlighting their potential contributions in the ongoing quest for sensitive markers of PD.

## Introduction

Given the high prevalence of frontostriatal motor disorders in general, and Parkinson’s disease (PD) in particular (Rossi et al., 2018), increasing efforts are being made to identify condition-sensitive markers (Delenclos et al., 2016). Cognitive evaluations prove highly useful, as they are inexpensive, non-invasive, and easily applicable (Bocanegra et al., 2015; García et al., 2017, 2018a). Frontostriatal circuits, which are crucially involved in motor function and become impaired early in PD (Samii et al., 2004; Rodriguez-Oroz et al., 2009), subserve multiple high-order functions, including decision-making, cognitive flexibility, attention, working memory, reward monitoring, motivation, error monitoring (Chudasama and Robbins, 2006; Morris et al., 2016; Birba et al., 2017), and, no less importantly, linguistic (Grossman et al., 2001; Ibáñez et al., 2013; Birba et al., 2017) and emotional (Takahashi et al., 2009; Baez et al., 2016, 2018) processing.

Candidate markers of frontostriatal disruptions have been obtained through separate assessments of specific verbal dimensions: syntax and emotional language processing (Paulmann et al., 2011; Bocanegra et al., 2015; Birba et al., 2017; Dissanayaka et al., 2017; García et al., 2017). Notwithstanding, most studies on PD have ignored the anatomical complexity and multifunctionality of frontostriatal circuits, considering language dimensions as compartmentalized (if not altogether modular) functions. This isolationist approach to cognitive processes precludes the identification of multidimensional markers, which are potentially more sensitive for the characterization and identification of PD patients. For instance, multidimensional linguistic (acoustic, prosodic, and semantic) markers surpass unidimensional ones in sorting between PD patients “on” and “off ” medication (Norel et al., 2018). Despite recent calls for more integrative multidimensional frameworks to characterize cognitive processes (Ibáñez and García, 2018; Ibáñez, 2019) and their dysfunctions in neurological conditions (Caselli et al., 2014; Canevelli et al., 2015; Delenclos et al., 2016), no study in PD has yet explored whether patient classification can be improved through a joint assessment of syntactic and emotional language processing. Therein lies the aim of this article.

As shown in multiple studies, frontostriatal compromise can be robustly indexed through performance on syntactic processing tasks (for a review, see Birba et al., 2017). Notably, subtle deficits have been obtained through assessments of functional-role assignment (a predominantly sequential form of syntactic processing) and the establishment of long-distance dependencies (which distinctly taxes hierarchical processing mechanisms; Bocanegra et al., 2015; García et al., 2017). In frontostriatal disorders like PD and Huntington’s disease (HD), these subdomains are affected in early stages irrespective of the patients’ executive skills or overall cognitive status (Bocanegra et al., 2015; García et al., 2018b). Moreover, evidence from asymptomatic PD-mutation carriers indicates that functional-role assignment can be selectively disrupted in preclinical stages, even before other linguistic or extralinguistic domains are affected (García et al., 2017). Therefore, performance on this particular dimension emerges as a potentially sensitive marker of the disease.

Also, frontostriatal atrophy has been linked to emotional processing (Baez et al., 2016, 2017, 2018). In particular, patients with PD show impairments in emotional language comprehension (Zgaljardic et al., 2003; Pell and Monetta, 2008). Furthermore, subtle impairments in motor disorders have been revealed through verbal measures of *Schadenfreude* (pleasure at others’ misfortunes). Response to sentences evoking *Schadenfreude* is selectively reduced upon frontostriatal atrophy (Baez et al., 2018). Alongside evidence of other emotional impairments in PD (Pell and Leonard, 2005; Ibarretxe-Bilbao et al., 2009), these findings suggest that verbal assessments of *Schadenfreude* could also reveal early deficits in this condition.

Notably, syntax and verbal emotion processing constitute different linguistic dimensions, characterized by dissimilar putative substrates [neostriatum for syntax (Szalisznyo et al., 2017), ventral striatum for *Schadenfreude* (Takahashi et al., 2009; Baez et al., 2018)], levels of automaticity [more automatic for syntax (Pulvermuller et al., 2008), more conscious for social emotions (Baez et al., 2017)], and connectivity patterns [increased connectivity between the striatum and Broca’s area for syntax (Teichmann et al., 2015), higher connectivity between the ventral striatum and insular regions for social emotions (Paulus et al., 2018)]. However, the evidence above indicates that, beyond their disparity, both domains are sensitive to subtle disturbances in early disease stages, which likely attests to the anatomical (Chudasama and Robbins, 2006), neurochemical (Chudasama and Robbins, 2006), and functional (Grossman et al., 2001; Morris et al., 2016) complexity of frontostriatal circuits affected in PD. This opens a fertile path for research, since the classification of patients with neurodegenerative disorders (Devanand et al., 2008), including PD (Delenclos et al., 2016; Norel et al., 2018), may be improved through multidimensional assessments.

Here, our assessment of syntax included functional-role assignment and long-distance dependencies tasks. For the assessment of emotional language processing, we focused on social emotions given that their ecological relevance to characterizing daily interpersonal skills (Baez et al., 2017). In the latter case, we employed a validated paradigm (Baez et al., 2016, 2018; Santamaria-Garcia et al., 2017; Gomez-Carvajal et al., 2020) consisting of declarative affirmative sentences, which trigger *Schadenfreude* and envy (another social emotion acting as a control condition). Considering previous evidence, we hypothesized that combined measures of functional-role assignment and *Schadenfreude* would yield better patient discrimination relative to other syntactic and emotional dimensions. Moreover, given that levodopa bioavailability has been shown to modulate performance in different linguistic (Herrera and Cuetos, 2012; Herrera et al., 2012) and emotional (Lawrence et al., 2007; Mondillon et al., 2012) tasks, we conducted an exploratory comparison between PD patients in “on” and “off ” stages of their medication (PD-on and PD-off, respectively). Briefly, this study aims to nurture an emergent trend highlighting the potential clinical benefits of multidimensional assessments for the classification of PD patients.

## Materials and Methods

### Participants

The study comprised 31 cognitively preserved PD patients and 24 healthy controls matched for age, sex, and years of education (Table 1). Patients were diagnosed according to the UK PD Society Brain Bank criteria (Hughes et al., 1992). Their motor symptoms were assessed with part III of the Unified Parkinson’s Disease Rating Scale (UPDRS) and the Hoehn & Yahr scale (H&Y). All patients completed this initial assessment in the “on” stage of Levodopa. Then, for our core language protocol, the PD sample was subdivided into patients tested “on” (*n* = 15) and “off ” (*n* = 16) medication. These subgroups were also paired in terms of age, sex, education, years since diagnosis, and UPDRS scores. To prevent biases in task administration, investigators were blinded to the patients’ medication status.

**Table 1:** Demographic and clinical characteristic of the participants.

	PD patients (*n* = 31) Mean (*SD*)	Controls (*n* = 24) Mean (*SD*)	PD-on (*n* = 15) Mean (*SD*)	PD-off (*n* = 16) Mean (*SD*)	PD vs. controls *p*-value	PD-on vs. PD-off *p*-value
**Demographics**
Age (years)^a^	61.74 (5.14)	59.58 (7.22)	61.20 (6.19)	62.25 (4.07)	0.20	0.57
Sex (F:M)^b^	13:18	12:12	6:9	7:9	0.55	0.83
Education (years)^a^	11.77 (4.16)	12.21 (4.40)	12.31 (3.83)	11.20 (4.55)	0.71	0.46
**Clinical assessment**
Years since diagnosis^a^	3.48 (1.48)	-	3.27 (1.39)	3.69 (1.59)	-	0.43
UPDRS-III^a^	18.68 (11.58)	-	21.93 (10.90)	15.63 (11.70)	-	0.13
L&B^a^	6.0 (1.48)	6.42 (1.56)	6.20 (1.52)	5.81 (1.47)	0.31	0.47
H&Y^a^	4.94 (3.08)	4.25 (3.14)	4.27 (2.82)	5.56 (3.27)	0.42	0.24
**Cognitive assessment**
MoCA^a^	25.0 (2.35)	25.38 (2.37)	25.0 (2.51)	25.0 (2.28)	0.56	1.00
IFS^a^	22.65 (3.70)	24.25 (3.09)	23.33 (3.92)	22.0 (3.48)	0.09	0.32

*PD, Parkinson’s disease; PD-on, Parkinson’s disease patients in the “on” state of medication; PD-off, Parkinson’s disease patients in the “off” state of medication; UPDRS, Unified Parkinson’s Disease Rating Scale; H&Y, Hoehn & Yahr Scale; L&B, Lawton and Brody Index; MoCA, Montreal Cognitive Assessment; IFS, INECO Frontal Screening battery. ^a^*p*-values were calculated through one-way ANOVA. ^b^*p*-values were calculated through the chi-squared test (*X*^2^). Alpha level set at 0.05*.

All samples were also comparable in terms of their independent living skills and depressive symptoms, as measured with Lawton and Brody Index (L&B) and the Hamilton Depression Rating Scale (HDRS), respectively. They were also matched for the general cognitive state, as assessed *via* the Montreal Cognitive Assessment (MoCA), and executive function skills, as measured with the INECO Frontal Screening (IFS). The MoCA (Nasreddine et al., 2005) comprises evaluates attention, executive functions, memory, language, visuoconstructional and visuospatial skills, conceptual thinking, calculations, and orientation. The IFS battery (Torralva et al., 2009) includes the following eight subtests: (1) motor programing (Luria series, “fist, edge, palm”); (2) conflicting instructions (hitting the table once when the administrator hits it twice, or hitting it twice when the administrator hits it only once); (3) motor inhibitory control; (4) numerical working memory (backward digit span); (5) verbal working memory (months backward); (6) spatial working memory (modified Corsi tapping test); (7) abstraction capacity (inferring the meaning of proverbs); and (8) verbal inhibitory control (modified Hayling test). Importantly, all of these tests have proven sensitive to frontostriatal disorders, including PD (Nazem et al., 2009; Bocanegra et al., 2015). See details in Table 1 and Supplementary Data 1, 2.

No subject in any group reported a history of alcohol/drug abuse, psychiatric conditions, or other neurological illnesses. All participants provided written consent in agreement with the Declaration of Helsinki. The Institutional Ethics Committee approved this study.

### Materials

#### Syntactic Tasks

Syntactic comprehension was examined through the Touching A with B and the Embedded Sentences subtests of the Boston Diagnostic Aphasia Examination (Goodglass et al., 2000), which are sensitive to frontostriatal disorders (García et al., 2018b), including PD (Bocanegra et al., 2015; García et al., 2017). In both subtests, participants were required to select which of four pictures best represents a given utterance read by the examiner. In Touching A with B (12 items, maximum score = 12), each picture depicts the hand of a person holding or touching objects. The examiner read sentences including the verb *touching* in present participle form and two nouns that vary in syntactic function. In some sentences, both nouns are the direct object of *touching* (e.g., *Touching the spoon and the scissors*), while, in others, one of the nouns is a direct object and the other is an instrumental adjunct (e.g., *Touching the scissors with the comb*). Therefore, this task taps the syntactic domain of functional-role assignment (García et al., 2017, 2018b). In the Embedded Sentences subtest (10 items, maximum score = 10), stimuli consist in sentences including a restrictive relative clause as part of their subject (e.g., *The woman who is fat is kissing her husband*) or direct object (e.g., *The girl is chasing the boy who is wearing boots*). Thus, this subtest focuses on the processing of long-distance dependencies (García et al., 2017, 2018b).

#### Socio-emotional Language Task

Levels of *Schadenfreude* and envy were measured with a verbal task that proves sensitive to frontostriatal disorders (Baez et al., 2016, 2018; Santamaria-Garcia et al., 2017). Participants were first shown a real-life photograph and a brief description of two characters matched in age and sex with each participant. Then, in the first experimental block, participants read 15 sentences describing fortunate situations occurring to either of the two characters, and they indicated how much envy they felt for the character on a scale from 1 (no envy) to 9 (extreme envy). In the second block, participants were presented with 15 unfortunate situations involving either character and they rated their levels of *Schadenfreude* from 1 (no pleasure) to 9 (extreme pleasure). Furthermore, five neutral events were included in each block for control purposes. Considering that envy predicts the levels of *Schadenfreude* (Takahashi et al., 2009), the envy block was presented first. Situations were pseudorandomly distributed within each block. See details in Supplementary Data 3.

All stimuli in the envy and *Schadenfreude* blocks consisted of declarative affirmative sentences, with their main verb in active voice and past tense (more precisely, *pretérito perfecto indefinido*). Also, all sentences in both sets comprised two clauses (standing in either paratactic or hypotactic relation) with a strictly systematic syntactic pattern [i.e., (tacit) subject + verb + optional complement].

### Statistical Analysis

Neuropsychological and behavioral data were analyzed using one-way ANOVAs. First, we compared the performance of all PD patients and all controls. Then, to assess the impact of medication state, we reiterated the analyses comparing PD-on vs. PD-off patients. Also, to control for the effect of general cognitive state, executive functions, and depressive symptoms on experimental results, we performed ANCOVA tests adjusted independently for total MoCA, IFS, and HDRS scores—for maximal informativeness, results are reported both before and after co-variation. Alpha levels were set at 0.05 for all analyses. Effect sizes were calculated through Cohen’s *d*, with cut-offs of 0.20, 0.50, and 0.80 for small, middle, and large effects, respectively.

Additionally, we performed multiple group discriminant function analyses (MDAs) to determine which measures best discriminate between: (a) PD patients and controls; and (b) PD-on and PD-off patients. In the first two MDAs, only those measures yielding between-group differences were considered as predictors. We then conducted a third MDA including both predictors together.

Moreover, two receiver-operating characteristics (ROC) curves were used to determine which of the measures showing between-group differences afforded the greatest sensitivity and specificity to discriminate between: (a) PD patients vs. controls; and (b) PD-on vs. PD-off patients. ROC curve analyses were performed using the variables yielding differences between PD patients and controls, first separately and then jointly. The areas under the ROC curves (AUCs; 95% CI) were used as the measure of discriminatory accuracy. Additionally, sensitivity and specificity were calculated.

Moreover, for exploratory purposes, we conducted MDA and ROC analyses to discriminate between PD-on and PD-off patients. Whereas inferential analyses can only reveal significant or non-significant effects at the *group* level, these approaches generate measures of classification accuracy, sensitivity, and specificity. Therefore, they reveal the *subject-level* probability with which patients can be identified as being on or off medication, shedding light on the role of dopamine bioavailability in syntax and emotional language processing.

## Results

### Syntactic Tasks

**Figure 1:**
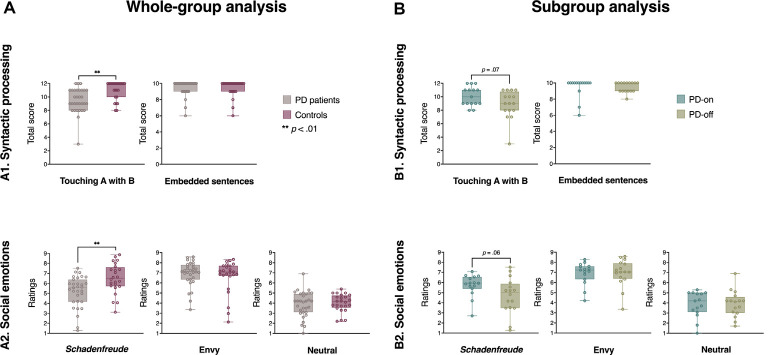
Group results from the syntactic and social emotion tasks. **(A)** Whole-group comparison between Parkinson’s disease (PD) patients and controls: **(A1)** syntactic processing scores; **(A2)** social emotion ratings. **(B)** Subgroup comparison between PD-on and PD-off patients: **(B1)** syntactic processing scores; **(B2)** social emotion ratings. Between-group comparisons were performed through one-way ANOVA.

Relative to controls, PD patients obtained significantly lower scores in Touching A with B (*F*_(1,53)_ = 10.81, *p* = 0.002, *d* = 0.91), but both groups performed similarly on the Embedded Sentences subtest (*F*_(1,53)_ = 1.48, *p* = 0.22, *d* = 0.35)—see Figure 1A1, and Supplementary Table 1. Significant differences between groups in Touching A with B were preserved after removing an outlier from the PD group (*F*_(1,52)_ = 10.75, *p* = 0.002, *d* = −0.89). Also, group differences in Touching A with B remained significant after co-varying for MoCA, IFS, and HDRS. Moreover, comparisons between PD-off and PD-on patients showed marginally poorer performance for the former on Touching A with B (*F*_(1,29)_ = 3.37, *p* = 0.07, *d* = 0.66), alongside non-significant differences on the Embedded Sentences subtest (*F*_(1,29)_ = 0.009, *p* = 0.92, *d* = 0.03)—see Figure 1B1, and Supplementary Table 1. The marginal differences between subgroups in Touching A with B remained similar after adjusting for MoCA, IFS, and HDRS.

### Socio-emotional Language Task

*Schadenfreude* ratings were lower in PD patients than in controls (*F*_(1,53)_ = 10.14, *p* = 0.002, *d* = 0.87), there being no significant between-group differences in ratings of envy (*F*_(1,53)_ = 0.61, *p* = 0.439, *d* = 0.21) and neutral situations (*F*_(1,53)_ = 0.18, *p* = 0.675, *d* = 0.12)—see Figure 1A2, and Supplementary Table 1. Significant differences between groups in *Schadenfreude* ratings were preserved after co-varying for MoCA, IFS, and HDRS. Also, *Schadenfreude* ratings were marginally lower for PD-off than PD-on patients (*F*_(1,29)_ = 3.65, *p* = 0.06, *d* = 0.69), but no differences emerged between these groups’ ratings of envy (*F*_(1,29)_ = 0.003, *p* = 0.955, *d* = 0.02) and neutral situations (*F*_(1,29)_ = 0.01, *p* = 0.910, *d* = 0.04)—see Figure 1B2, and Supplementary Table 1. Such marginal differences between subgroups in *Schadenfreude* ratings remained similar after co-variation with MoCA, IFS, and HDRS scores.

### MDA Analyses

#### MDA Between PD Patients and Controls

**Figure 2:**
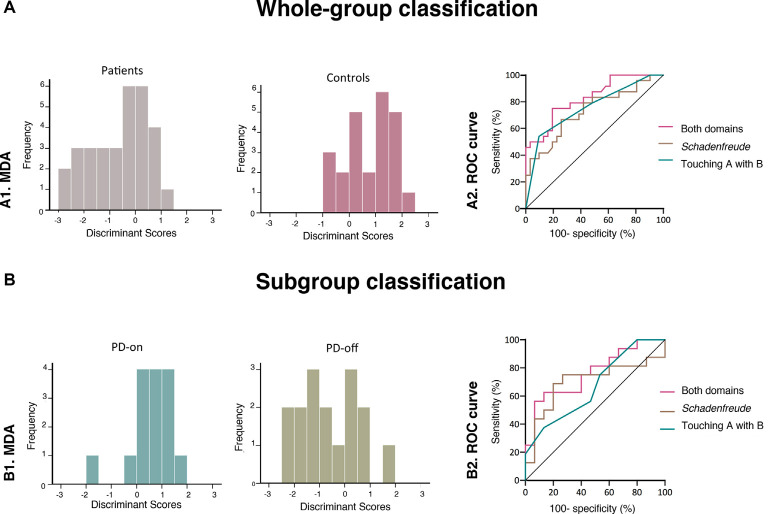
Subject classification results. **(A)** Whole-group classification between PD patients and controls: **(A1)** histograms showing the distribution of Touching A with B and Schadenfreude discriminating scores; **(A2)** receiver-operating characteristics (ROC) curves for Touching A with B scores, Schadenfreude ratings, and the combination of both domains. **(B)** Subgroup classification between PD-on and PD-off patients: **(B1)** histograms showing the distribution of Touching A with B and Schadenfreude discriminating scores; **(B2)** ROC curves for Touching A with B scores, Schadenfreude ratings, and combined outcomes from both domains. Classification accuracies were calculated through the ROC curve and multiple group discriminant function analyses.

Including the Touching A with B score as predictor, we obtained one discriminant function with a Wilkis’s *λ* = 0.831, $\chi_{\left(1\right)}^{2}$ = 9.741, *p* = 0.002. This function correctly classified 67.3% of the cases (64.5% of PD patients and 70.8% of controls). Then, using *Schadenfreude* ratings as predictor, we obtained one discriminant function with a Wilkis’s *λ* = 0.8.39, $\chi_{\left(1\right)}^{2}$ = 9.192, *p* = 0.002. This function classified 63.6% of the cases into their respective groups (58.1% of PD patients and 70.8% of controls). Finally, when both domains were introduced as predictors, we obtained one discriminant function with a Wilkis’s *λ* = 0.684, $X_{\left(2\right)}^{2}$ = 19.712, *p* < 0.001. The Touching A with B total score discriminated most reliably between PD patients and controls, followed by the *Schadenfreude* ratings. This function accounted for 100% of the total variance. This model showed the best classification accuracy across all three MDAs, successfully classifying 70.9% of the participants (67.7% of PD patients and 75.0% of controls)—Figure 2A1. Standardized coefficients of predictors included in each MDA are shown in Supplementary Table 2.

#### MDA Between PD-On and PD-Off Patients

Entering the Touching A with B score as predictor, we attained one discriminant function with a Wilkis’s *λ* = 0.896, $X_{\left(1\right)}^{2}$ = 3.131, *p* = 0.07. This function classified 54.8% of the cases into their actual group (53.3% of PD-on and 56.3% of PD-off patients). Then using *Schadenfreude* ratings as predictor, we obtained one discriminant function with a Wilkis’s *λ* = 0.888, $X_{\left(1\right)}^{2}$ = 3.383, *p* = 0.06. This function classified 64.5% of the cases into their corresponding group (80.0% of PD-on and 50.0% of PD-off patients). Finally, when both domains were included as predictors, one discriminant function was calculated with a Wilkis’s *λ* = 0.762, $X_{\left(2\right)}^{2}$ = 7.611, *p* = 0.02. *Schadenfreude* ratings showed the best discrimination accuracy, followed by the Touching A with a B score. This function accounted for 100% of the total variance and showed the best classification accuracy, correctly classifying 74.2% of the cases (86.7% of PD-on and 62.5% of PD-off patients)—see Figure 2B1. Standardized coefficients of predictors included in each MDA are shown in Supplementary Table 2.

### ROC Curve Analyses

#### ROC Curve Analysis Between PD Patients and Controls

At a cut-off of 11.5 points, Touching A with B scores yielded a sensitivity of 54.2% and a specificity of 90.3%. The AUC was 0.76 (CI: 0.62–0.89; *p* = 0.001). Then, at a cut-off of six points, *Schadenfreude* ratings showed a sensitivity of 66.7% and a specificity of 74.2%. The AUC was 0.73 (CI: 0.59–0.87; *p* = 0.003). The average of both domains showed the best discriminatory accuracy, reaching a sensitivity of 75% and a specificity of 80.7% at a cut-off of 8.3 points. The AUC was 0.83 (CI: 0.72–0.94; *p* < 0.001). The ROC curves for the three variables are illustrated in Figure 2A2.

#### ROC Curve Analysis Between PD-On and PD-Off Patients

At a cut-off score of 9.5 points, Touching A with B score showed a sensitivity of 56.3% and a specificity of 53.3%. The AUC was 0.67 (CI: 0.48–0.86; *p* = 0.10). Then, at a cut-off of 5.5 points, *Schadenfreude* ratings showed a sensitivity of 68.8% and a specificity of 73.3%. The AUC was 0.73 (CI: 0.50–0.90; *p* = 0.05). The average of both domains afforded the highest discriminatory accuracy, reaching a sensitivity of 62.5% and a specificity of 66.7% at a cut-off of 7.5 points. The AUC was 0.76 (CI: 0.59–0.93; *p* = 0.01). The ROC curves for the three variables are illustrated in Figure 2B2.

## Discussion

This report documents the first joint evaluation of two linguistic domains relying on frontostriatal circuits affected in PD: syntactic and verbal emotional processing. Patients exhibited selective impairments in specific measures of each dimension (functional-role assignment and *Schadenfreude*, respectively). More crucially, results from two analytical approaches showed that individual patient classification improved when combining outcomes from both dimensions. These findings suggest that multidimensional linguistic assessments may better capture the complex and multifunctional impact of frontostriatal disruptions.

Frontostriatal disorders have been shown to impair syntactic comprehension and syntactic judgment skills (Bocanegra et al., 2015; García et al., 2018b; Johari et al., 2019; Whitfield and Gravelin, 2019; Melchionda et al., 2020). Here, we found that PD patients were impaired in Touching A with B but not in the Embedded Sentences subtest. This very dissociation has been observed in persons at risk for PD, even before the onset of other linguistic, cognitive, or motor impairments (García et al., 2017). As noted elsewhere (García et al., 2017, 2018b), the Touching A with B test taps functional-role assignment, a skill that rests mainly on sequential (as opposed to hierarchical) syntactic processes. In line with computational works suggesting that different sub-portions of the striatum play distinct roles during linguistic processing (Szalisznyo et al., 2017), this selective pattern might be partially explained by the nigral origins of frontostriatal deficits in PD (Birba et al., 2017). Also, this deficit was not associated with the patients’ general cognitive state, executive skills, or depression symptoms. Such a result suggests that functional-role assignment deficits in PD may represent a primary dysfunction, rather than a secondary manifestation of unspecific cognitive/affective alterations. Still, further research is needed to clarify the role of different frontostriatal pathways in specific syntactic domains.

Regarding socio-emotional processing, PD patients reported lower levels of *Schadenfreude* than controls, despite null differences in ratings of envy and neutral situations. As was the case with syntactic outcomes, this pattern was not associated with the patients’ overall cognitive status, executive functions, or depression symptoms, attesting to its potential primary (rather than epiphenomenal) nature. Our findings replicate findings from other frontostriatal disorders, such as HD (Baez et al., 2016, 2018). This attests to the intimate link between such circuits and this particular social emotion (Takahashi et al., 2009; Baez et al., 2018) as well as its underlying operations, such as reward processing and mentalizing abilities (Takahashi et al., 2009; Poletti et al., 2011). Those two operations are impaired in PD (Schott et al., 2007; Poletti et al., 2011), suggesting that the sensitivity of *Schadenfreude* as a marker of frontostriatal abnormalities might rest on multi-determined neurocognitive foundations. In particular, *Schadenfreude* levels have been associated with increased activity in the ventral striatum activity in healthy participants (Takahashi et al., 2009) and ventral striatum gray matter reduction in frontostriatal disorders (Baez et al., 2018). Reduced dopamine transporter density (Remy et al., 2005; Cilia et al., 2010) and reduced activity (Rao et al., 2010) in the ventral striatum have been previously reported in patients with PD. These functional abnormalities may underlie reduced *Schadenfreude* levels observed in PD patients. As a recent study (Multani et al., 2019) reported associations between increased functional connectivity between opercular and insular cortices and socio-emotional processing in PD, future studies should investigate the structural and functional brain correlates (beyond frontostriatal pathways) of socio-emotional language processing in PD.

Interestingly, performance on Touching A with B tended to be poorer in PD-off relative to PD-on patients, there being no significant differences between such subgroups in the Embedded Sentences subtest. Tentatively, early deficits in the functional-role assignment may be associated not only with frontostriatal atrophy but also with dopamine bioavailability, as seen in other linguistic domains. PD-off patients exhibit more difficulties than PD-on patients in picture naming (Herrera and Cuetos, 2012), phonological and action fluency (Herrera et al., 2012), and sentence comprehension (Grossman et al., 2001) tasks. Also, we found marginally higher *Schadenfreude* ratings in PD-on compared to PD-off patients. Though not focused on *Schadenfreude*, previous studies suggest that dopamine therapy increases emotion recognition in PD (Dujardin et al., 2004; Mondillon et al., 2012; Dan et al., 2019). As stated above, *Schadenfreude* has been liked to ventral striatum activity and volume (Takahashi et al., 2009; Baez et al., 2018). Also, dopamine supplementation seems to improve functions mediated by dorsal striatum and to modulate ventral-striatal operations (Gotham et al., 1988; Kish et al., 1988; Macdonald and Monchi, 2011). Briefly, although present results should be taken with reservation given the moderate size of each patient subgroup, they invite new specific studies aimed to assess the role of dopamine in syntax and emotional language processing.

Yet, beyond those individual patterns, our core finding is that patient classification was boosted upon joint analysis of these sensitive measures. Specifically, an MDA including both dimensions successfully classified 70.9% of the participants while individual measures of functional-role assignment and *Schadenfreude* reached accuracies of 67.3% and 63.6%, respectively. Furthermore, ROC curves for the combination of both measures increased sensitivity and specificity values. Similarly, MDA and ROC analyses also showed that a combination of such measures improved classification between PD-on vs. PD-off patients. Taken together, these results suggest that multidimensional assessments can better capture the high complexity of frontostriatal networks, whose widespread anatomical distribution (Chudasama and Robbins, 2006), varied neurochemical dynamics (Chudasama and Robbins, 2006), and multiple connectivity patterns (Morris et al., 2016) render them putatively involved in diverse higher-order domains cutting across multiple subfunctions.

Note that similar classification accuracies have been reported by previous studies using cognitive measures in PD and other neurodegenerative diseases (Bennett et al., 2006; García et al., 2016; Tkaczynska et al., 2020). Indeed, our classification results are even higher than those of a recent study (Zimmerer et al., 2020) using linguistic measures to classify syndromes which primarily impair language (i.e., primary progressive aphasia). In line with previous results (Norel et al., 2018), our findings suggest that the joint assessment of different linguistic skills can boost the detection of PD cases, as observed for other domains in different neurodegenerative disorders (Caselli et al., 2014). Still, these outcomes do not yet warrant direct testing of our tools’ clinical applicability. Rather, they lay the groundwork for more extensive research testing the translational utility of multidimensional assessments, in line with recent calls to validate inexpensive, non-invasive, patient-friendly markers of PD and other conditions (Canevelli et al., 2015; Delenclos et al., 2016).

Similarly, joint consideration of both dimensions also improved the classification of PD-on vs. PD-off patients, reaching an accuracy of 74.2%. However, the classification of PD-on patients (82.7%) was better than that of PD-off patients (62.5%). This probably reflects the multivariate nature of the MDA method, which combines independent variables to classify participants in different groups according to discriminant scores of selected predictors (Stevens, 2002). The cases are assigned to groups based on their discriminant scores and an appropriate decision rule. For example, in two-group discriminant analysis, a case will be assigned to the group whose centroid (the mean values for the discriminant scores for a particular group) is the closest. The fact that PD-off had worse classification than PD-on means that, in some PD-off patients, Touching A with B and *Schadenfreude* outcomes were similar to those of PD-on patients. This finding may be influenced by two factors. First, neuropsychological and clinical heterogeneity is a central characteristic of PD (Kehagia et al., 2010). Given that we used a between-group design, this heterogeneity could be reflected differently in either the PD-on or the PD-off groups. Second, the role of Levodopa withdrawal on syntax and *Schadenfreude* measures has not been established. Although some studies suggest that PD-off show lower performance than PD-on patients in syntax (Grossman et al., 2001) and emotion processing (Dujardin et al., 2004; Dan et al., 2019), others reported a comparable deficit in patients whatever the treatment condition (Sprengelmeyer et al., 2003). Our results suggest that scores in Touching A with B and *Schadenfreude* measures are lower among PD-off patients, but some of these patients performed similarly to those in the PD-on group. This heterogeneity among patients in the PD-off group could be associated with several individual factors such as disease severity (MacDonald et al., 2013) and levels of apathy or depression (Cohen et al., 2015). Future studies using larger samples of PD-on and PD-off patients should further investigate the role of dopamine withdrawal on linguistic and emotional domains, and the association of disease severity and neuropsychiatric symptoms on Levodopa response.

Despite differences in discrimination accuracy between PD-on and PD-off patients, overall, our results suggest that performance in syntactic and emotional language processing could be associated with dopamine bioavailability. Considering that ANOVAs failed to reveal significant differences between such groups, this finding carries a non-trivial methodological implication: estimations of subject-level classification probabilities may offer useful insights irrespective of group-level results. Indeed, a previous study assessing linguistic measures failed to find significant differences between PD patients and controls but showed that grammatical and sematic patterns identified in monologues accurately discriminated between groups (García et al., 2016). Still, the association between dopamine bioavailability and performance in the syntactic and emotional language in PD should be more deeply assessed in future studies.

More generally, our results have theoretical implications. First, traditional frameworks in neuroscience and neuropsychology often favor rather modular accounts of particular linguistic domains. However, in daily interactions, different linguistic processes are intertwined and automatically interconnected with each other and with several other cognitive, affective, motoric, and even interoceptive functions (Ibáñez, 2019). The current multidimensional approach represents a viable approximation to circumvent such counterfactual ethos, in line with recent calls (Ibáñez and García, 2018; Ibáñez, 2019) for a more ecological, dynamic, and synergetic view of cognitive processes. Our results support novel frameworks pinpointing the multiple non-motor functions of the basal ganglia, crucially including linguistic and emotional processing (Eisinger et al., 2018). Accordingly, this work incarnates a concrete implementation of the emergent intercognitive agenda (Ibáñez, 2019) as an avenue towards more sophisticated conceptions of human cognition (Ibáñez and García, 2018).

Also, our results pave the way for developing multidimensional cognitive assessments to characterize and identify early PD patients, as highlighted in recent works (Canevelli et al., 2015; Delenclos et al., 2016). Such assessments may afford potential cognitive markers for detecting and tracking the progression of PD or other frontostriatal disorders, offering more robust approximations to the anatomical complexity and multifunctionality of frontostriatal circuits (Birba et al., 2017). Future studies should further investigate the potential use of combining linguistic and otherwise cognitive measures for early and preclinical PD detection. This is consistent with a recent theoretical perspective (Morese and Palermo, 2020) proposing an interdisciplinary vision in PD to encourage a richer discussion capable of generating new research and developing interventions to improve social and cognitive functioning in PD patients. Furthermore, as the results of a previous study in PD animal models (Ztaou et al., 2018) highlighted the relevance of striatal cholinergic interneurons in emotional and other non-motor deficits, future studies should also assess the role of cholinergic medication on emotional language processing in PD patients.

Some limitations of our work should be acknowledged. First, our sample size was relatively small. However, it proved similar to that of previous studies on linguistic (Grossman et al., 2000, 2002, 2003; Angwin et al., 2005, 2007; Bocanegra et al., 2015) and emotional (Breitenstein et al., 2001; Dara et al., 2008; Martínez-Corral et al., 2010) dimensions in PD. Future studies assessing PD patients with multidimensional assessments should include larger sample sizes. Second, we compared PD-on vs. PD-off patients using a between-subjects design. Future research should explore the role of dopamine medication using within-subject designs. Finally, as we did not include neuroimaging measures, our interpretations of the associations between the pathogenesis of PD and Touching A with B and *Schadenfreude* scores are hypothetical. Further research is needed to understand the complex relationship between frontostriatal pathways functioning in PD and different linguistic and emotional dimensions.

In sum, our study indicates that a joint evaluation of syntactic and socio-emotional language tasks can improve the classification accuracy of early PD patients. This result informs an emergent trend emphasizing the relevance of multidimensional cognitive examinations across frontostriatal disorders. Looking forward, new applications of this approach should be implemented to boost the ongoing quest for early markers of these conditions.

## Data Availability Statement

The raw data supporting the conclusions of this article will be made available by the authors, without undue reservation.

## Ethics Statement

The studies involving human participants were reviewed and approved by Ethics Committee of the Icesi University. The patients/participants provided their written informed consent to participate in this study.

## Author Contributions

SB, EH, HS-G, AI, and AG developed the study concept and the study design. EH, JC, and MP performed testing and data collection. SB, CT, and HS-G performed the data analysis and interpretation under the supervision of AI and AG. SB, EH, CT, and JC drafted the manuscript. MP, HS-G, AI, and AG provided critical revisions. All authors contributed to the article and approved the submitted version.

## Conflict of Interest

The authors declare that the research was conducted in the absence of any commercial or financial relationships that could be construed as a potential conflict of interest.

## References

[B1] Angwin A. J. Chenery H. J. Copland D. A. Murdoch B. E. Silburn P. A. (2005). Summation of semantic priming and complex sentence comprehension in Parkinson’s disease. Cogn. Brain Res. 25, 78–89. 10.1016/j.cogbrainres.2005.04.008 15894470

[B2] Angwin A. J. Chenery H. J. Copland D. A. Murdoch B. E. Silburn P. A. (2007). The speed of lexical activation is altered in Parkinson’s disease. J. Clin. Exp. Neuropsychol. 29, 73–85. 10.1080/13803390500507188 17162724

[B3] Baez S. García A. M. Santamaria-Garcia H. (2017). “Moral cognition and moral emotions,” in Neuroscience and Social Science: The Missing Link, eds Ibanez A. Sedeno L. García A. M. (Cham: Springer Nature).

[B4] Baez S. Pino M. Berrio M. Santamaria-Garcia H. Sedeno L. Garcia A. M.. (2018). Corticostriatal signatures of schadenfreude: evidence from Huntington’s disease. J. Neurol. Neurosurg. Psychiatry 89, 112–116. 10.1136/jnnp-2017-316055 28765320

[B5] Baez S. Santamaria-Garcia H. Orozco J. Fittipaldi S. Garcia A. M. Pino M.. (2016). Your misery is no longer my pleasure: reduced schadenfreude in Huntington’s disease families. Cortex 83, 78–85. 10.1016/j.cortex.2016.07.009 27498039

[B6] Bennett I. J. Golob E. J. Parker E. S. Starr A. (2006). Memory evaluation in mild cognitive impairment using recall and recognition tests. J. Clin. Exp. Neuropsychol. 28, 1408–1422. 10.1080/13803390500409583 17050267

[B7] Birba A. Garcia-Cordero I. Kozono G. Legaz A. Ibanez A. Sedeno L.. (2017). Losing ground: frontostriatal atrophy disrupts language embodiment in Parkinson’s and Huntington’s disease. Neurosci. Biobehav. Rev. 80, 673–687. 10.1016/j.neubiorev.2017.07.011 28780312

[B8] Bocanegra Y. Garcia A. M. Pineda D. Buritica O. Villegas A. Lopera F.. (2015). Syntax, action verbs, action semantics and object semantics in Parkinson’s disease: dissociability, progression and executive influences. Cortex 69, 237–254. 10.1016/j.cortex.2015.05.022 26103601

[B10] Breitenstein C. Van Lancker D. Daum I. Waters C. H. (2001). Impaired perception of vocal emotions in Parkinson’s disease: influence of speech time processing and executive functioning. Brain Cogn. 45, 277–314. 10.1006/brcg.2000.1246 11237372

[B11] Canevelli M. Blasimme A. Vanacore N. Bruno G. Cesari M. (2015). From evidence to action: promoting a multidimensional approach to mild cognitive impairment. J. Am. Med. Dir. Assoc. 16, 710–711. 10.1016/j.jamda.2015.04.013 26027720

[B12] Caselli R. J. Locke D. E. Dueck A. C. Knopman D. S. Woodruff B. K. Hoffman-Snyder C.. (2014). The neuropsychology of normal aging and preclinical Alzheimer’s disease. Alzheimers Dement. 10, 84–92. 10.1016/j.jalz.2013.01.004 23541188 PMC3700591

[B13] Chudasama Y. Robbins T. W. (2006). Functions of frontostriatal systems in cognition: comparative neuropsychopharmacological studies in rats, monkeys and humans. Biol. Psychol. 73, 19–38. 10.1016/j.biopsycho.2006.01.005 16546312

[B14] Cilia R. Ko J. H. Cho S. S. van Eimeren T. Marotta G. Pellecchia G.. (2010). Reduced dopamine transporter density in the ventral striatum of patients with Parkinson’s disease and pathological gambling. Neurobiol. Dis. 39, 98–104. 10.1016/j.nbd.2010.03.013 20338240

[B15] Cohen M. L. Aita S. Mari Z. Brandt J. (2015). The unique and combined effects of apathy and depression on cognition in Parkinson’s disease. J. Parkinsons Dis. 5, 351–359. 10.3233/jpd-140484 25769262

[B16] Dan R. Ružička F. Bezdicek O. Roth J. Ružička E. Vymazal J.. (2019). Impact of dopamine and cognitive impairment on neural reactivity to facial emotion in Parkinson’s disease. Eur. Neuropsychopharmacol. 29, 1258–1272. 10.1016/j.euroneuro.2019.09.003 31607424

[B17] Dara C. Monetta L. Pell M. D. (2008). Vocal emotion processing in Parkinson’s disease: reduced sensitivity to negative emotions. Brain Res. 1188, 100–111. 10.1016/j.brainres.2007.10.034 18022608

[B18] Delenclos M. Jones D. R. McLean P. J. Uitti R. J. (2016). Biomarkers in Parkinson’s disease: advances and strategies. Parkinsonism Relat. Disord. 22, S106–S110. 10.1016/j.parkreldis.2015.09.048 26439946 PMC5120398

[B19] Devanand D. P. Liu X. Tabert M. H. Pradhaban G. Cuasay K. Bell K.. (2008). Combining early markers strongly predicts conversion from mild cognitive impairment to Alzheimer’s disease. Biol. Psychiatry 64, 871–879. 10.1016/j.biopsych.2008.06.020 18723162 PMC2613777

[B20] Dissanayaka N. N. W. Au T. R. Angwin A. J. O’sullivan J. D. Byrne G. J. Silburn P. A.. (2017). N400 and emotional word processing in Parkinson’s disease. Neuropsychology 31, 585–595. 10.1037/neu0000333 28287777

[B22] Dujardin K. Blairy S. Defebvre L. Duhem S. Noël Y. Hess U.. (2004). Deficits in decoding emotional facial expressions in Parkinson’s disease. Neuropsychologia 42, 239–250. 10.1016/s0028-3932(03)00154-4 14644109

[B23] Eisinger R. S. Urdaneta M. E. Foote K. D. Okun M. S. Gunduz A. (2018). Non-motor characterization of the basal ganglia: evidence from human and non-human primate electrophysiology. Front. Neurosci. 12:385. 10.3389/fnins.2018.00385 30026679 PMC6041403

[B24] García A. M. Bocanegra Y. Herrera E. Moreno L. Carmona J. Baena A.. (2018a). Parkinson’s disease compromises the appraisal of action meanings evoked by naturalistic texts. Cortex 100, 111–126. 10.1016/j.cortex.2017.07.003 28764852

[B25] García A. M. Bocanegra Y. Herrera E. Pino M. Munoz E. Sedeno L.. (2018b). Action-semantic and syntactic deficits in subjects at risk for Huntington’s disease. J. Neuropsychol. 12, 389–408. 10.1111/jnp.12120 28296213

[B26] García A. M. Carrillo F. Orozco-Arroyave J. R. Trujillo N. Vargas Bonilla J. F. Fittipaldi S.. (2016). How language flows when movements don’t: an automated analysis of spontaneous discourse in Parkinson’s disease. Brain Lang. 162, 19–28. 10.1016/j.bandl.2016.07.008 27501386

[B27] García A. M. Sedeno L. Trujillo N. Bocanegra Y. Gomez D. Pineda D.. (2017). Language deficits as a preclinical window into Parkinson’s disease: evidence from asymptomatic parkin and dardarin mutation carriers. J. Int. Neuropsychol. Soc. 23, 150–158. 10.1017/S1355617716000710 28205494

[B28] Gomez-Carvajal A. M. Santamaria-Garcia H. Garcia A. M. Valderrama M. Mejia J. Santamaria-Garcia J.. (2020). The unique social sense of puerperium: increased empathy and schadenfreude in parents of newborns. Sci. Rep. 10:5760. 10.1038/s41598-020-62622-7 32238840 PMC7113282

[B29] Goodglass H. Kaplan E. Barresi B. (2000). The Assessment of Aphasia and Related Disorders 3rd Edition. Philadelphia, PA: Lippincott Williams & Wilkins.

[B30] Gotham A. M. Brown R. G. Marsden C. D. (1988). ‘Frontal’ cognitive function in patients with Parkinson’s disease ‘on’ and ‘off’ levodopa. Brain 111, 299–321. 10.1093/brain/111.2.299 3378138

[B31] Grossman M. Cooke A. DeVita C. Lee C. Alsop D. Detre J.. (2003). Grammatical and resource components of sentence processing in Parkinson’s disease: an fMRI study. Neurology 60, 775–781. 10.1212/01.wnl.0000044398.73241.13 12629232

[B32] Grossman M. Glosser G. Kalmanson J. Morris J. Stern M. B. Hurtig H. I. (2001). Dopamine supports sentence comprehension in Parkinson’s disease. J. Neurol. Sci. 184, 123–130. 10.1016/s0022-510x(00)00491-3 11239945

[B33] Grossman M. Kalmanson J. Bernhardt N. Morris J. Stern M. B. Hurtig H. I. (2000). Cognitive resource limitations during sentence comprehension in Parkinson’s disease. Brain Lang. 73, 1–16. 10.1006/brln.2000.2290 10872635

[B34] Grossman M. Zurif E. Lee C. Prather P. Kalmanson J. Stern M. B.. (2002). Information processing speed and sentence comprehension in Parkinson’s disease. Neuropsychology 16, 174–181. 10.1037/0894-4105.16.2.174 11949709

[B35] Herrera E. Cuetos F. (2012). Action naming in Parkinson’s disease patients on/off dopamine. Neurosci. Lett. 513, 219–222. 10.1016/j.neulet.2012.02.045 22387157

[B36] Herrera E. Cuetos F. Ribacoba R. (2012). Verbal fluency in Parkinson’s disease patients on/off dopamine medication. Neuropsychologia 50, 3636–3640. 10.1016/j.neuropsychologia.2012.09.016 22995942

[B37] Hughes A. J. Daniel S. E. Kilford L. Lees A. J. (1992). Accuracy of clinical diagnosis of idiopathic Parkinson’s disease: a clinico-pathological study of 100 cases. J. Neurol. Neurosurg. Psychiatry 55, 181–184. 10.1136/jnnp.55.3.181 1564476 PMC1014720

[B38] Ibáñez A. (2019). Insular networks and intercognition in the wild. Cortex 115, 341–344. 10.1016/j.cortex.2019.01.024 30808551

[B40] Ibáñez A. Cardona J. F. Dos Santos Y. V. Blenkmann A. Aravena P. Roca M.. (2013). Motor-language coupling: direct evidence from early Parkinson’s disease and intracranial cortical recordings. Cortex 49, 968–984. 10.1016/j.cortex.2012.02.014 22482695

[B39] Ibáñez A. García A. M. (Eds) (2018). “The forest behind (and beyond) the trees,” in Contextual Cognition: The Sensus Communis of a Situated Mind (Springer International Publishing), 55–72.

[B41] Ibarretxe-Bilbao N. Junque C. Tolosa E. Marti M. J. Valldeoriola F. Bargallo N. (2009). Neuroanatomical correlates of impaired decision-making and facial emotion recognition in early Parkinson’s disease. Eur. J. Neurosci. 30, 1162–1171. 10.1111/j.1460-9568.2009.06892.x 19735293

[B42] Johari K. Walenski M. Reifegerste J. Ashrafi F. Behroozmand R. Daemi M.. (2019). A dissociation between syntactic and lexical processing in Parkinson’s disease. J. Neurolinguistics 51, 221–235. 10.1016/j.jneuroling.2019.03.004 31777416 PMC6880793

[B43] Kehagia A. A. Barker R. A. Robbins T. W. (2010). Neuropsychological and clinical heterogeneity of cognitive impairment and dementia in patients with Parkinson’s disease. Lancet Neurol. 9, 1200–1213. 10.1016/S1474-4422(10)70212-X 20880750

[B44] Kish S. J. Shannak K. Hornykiewicz O. (1988). Uneven pattern of dopamine loss in the striatum of patients with idiopathic Parkinson’s disease. Pathophysiologic and clinical implications. N. Engl. J. Med. 318, 876–880. 10.1056/nejm198804073181402 3352672

[B45] Lawrence A. D. Goerendt I. K. Brooks D. J. (2007). Impaired recognition of facial expressions of anger in Parkinson’s disease patients acutely withdrawn from dopamine replacement therapy. Neuropsychologia 45, 65–74. 10.1016/j.neuropsychologia.2006.04.016 16780901

[B47] Macdonald P. A. Monchi O. (2011). Differential effects of dopaminergic therapies on dorsal and ventral striatum in Parkinson’s disease: implications for cognitive function. Parkinsons Dis. 2011:572743. 10.4061/2011/572743 21437185 PMC3062097

[B46] MacDonald A. A. Monchi O. Seergobin K. N. Ganjavi H. Tamjeedi R. MacDonald P. A. (2013). Parkinson’s disease duration determines effect of dopaminergic therapy on ventral striatum function. Mov. Disord. 28, 153–160. 10.1002/mds.25152 23165957

[B48] Martínez-Corral M. Pagonabarraga J. Llebaria G. Pascual-Sedano B. García-Sánchez C. Gironell A.. (2010). Facial emotion recognition impairment in patients with Parkinson’s disease and isolated apathy. Parkinsons Dis. 2010:930627. 10.4061/2010/930627 20976097 PMC2957329

[B49] Melchionda D. Varvara G. Perfetto D. Mascolo B. Avolio C. (2020). Perceptive and subjective evaluation of speech disorders in Parkinson’s disease. J. Biol. Regul. Homeost. Agents 34, 683–686. 10.23812/19-412-L-2 32475102

[B50] Mondillon L. Mermillod M. Musca S. C. Rieu I. Vidal T. Chambres P.. (2012). The combined effect of subthalamic nuclei deep brain stimulation and L-dopa increases emotion recognition in Parkinson’s disease. Neuropsychologia 50, 2869–2879. 10.1016/j.neuropsychologia.2012.08.016 22944002

[B51] Morese R. Palermo S. (2020). Altruistic punishment and impulsivity in parkinson’s disease: a social neuroscience perspective. Front. Behav. Neurosci. 14:102. 10.3389/fnbeh.2020.00102 32792921 PMC7385270

[B52] Morris L. S. Kundu P. Dowell N. Mechelmans D. J. Favre P. Irvine M. A.. (2016). Fronto-striatal organization: defining functional and microstructural substrates of behavioural flexibility. Cortex 74, 118–133. 10.1016/j.cortex.2015.11.004 26673945 PMC4729321

[B53] Multani N. Taghdiri F. Anor C. J. Varriano B. Misquitta K. Tang-Wai D. F.. (2019). Association between social cognition changes and resting state functional connectivity in frontotemporal dementia, Alzheimer’s disease, Parkinson’s disease and healthy controls. Front. Neurosci. 13:1259. 10.3389/fnins.2019.01259 31824254 PMC6883726

[B54] Nasreddine Z. S. Phillips N. A. Bedirian V. Charbonneau S. Whitehead V. Collin I.. (2005). The montreal cognitive assessment, MoCA: a brief screening tool for mild cognitive impairment. J. Am. Geriatr. Soc. 53, 695–699. 10.1111/j.1532-5415.2005.53221.x 15817019

[B55] Nazem S. Siderowf A. D. Duda J. E. Have T. T. Colcher A. Horn S. S. (2009). Montreal cognitive assessment performance in patients with Parkinson’s disease with “normal” global cognition according to mini-mental state examination score. J. Am. Geriatr. Soc. 57, 304–308. 10.1111/j.1532-5415.2008.02096.x 19170786 PMC2754699

[B56] Norel R. Agurto C. Rice J. J. Ho B. K. Cecchi G. A. (2018). Speech-based identification of L-DOPA ON/OFF state in Parkinson’s disease subjects. bioRxiv [Preprint]. 10.1101/420422

[B57] Paulmann S. Ott D. V. Kotz S. A. (2011). Emotional speech perception unfolding in time: the role of the basal ganglia. PLoS One 6:e17694. 10.1371/journal.pone.0017694 21437277 PMC3060083

[B58] Paulus F. M. Muller-Pinzler L. Stolz D. S. Mayer A. V. Rademacher L. Krach S. (2018). Laugh or cringe? Common and distinct processes of reward-based schadenfreude and empathy-based fremdscham. Neuropsychologia 116, 52–60. 10.1016/j.neuropsychologia.2017.05.030 28583386

[B59] Pell M. D. Leonard C. L. (2005). Facial expression decoding in early Parkinson’s disease. Cogn. Brain Res. 23, 327–340. 10.1016/j.cogbrainres.2004.11.004 15820640

[B60] Pell M. D. Monetta L. (2008). How Parkinson’s disease affects nonverbal communication and language processing. Lang. Linguist. Compass 2, 739–759. 10.1111/j.1749-818X.2008.00074.x

[B61] Poletti M. Enrici I. Bonuccelli U. Adenzato M. (2011). Theory of mind in Parkinson’s disease. Behav. Brain Res. 219, 342–350. 10.1016/j.bbr.2011.01.010 21238496

[B62] Pulvermuller F. Shtyrov Y. Hasting A. S. Carlyon R. P. (2008). Syntax as a reflex: neurophysiological evidence for early automaticity of grammatical processing. Brain Lang. 104, 244–253. 10.1016/j.bandl.2007.05.002 17624417

[B63] Rao H. Mamikonyan E. Detre J. A. Siderowf A. D. Stern M. B. Potenza M. N.. (2010). Decreased ventral striatal activity with impulse control disorders in Parkinson’s disease. Mov. Disord. 25, 1660–1669. 10.1002/mds.23147 20589879 PMC3063061

[B64] Remy P. Doder M. Lees A. Turjanski N. Brooks D. (2005). Depression in Parkinson’s disease: loss of dopamine and noradrenaline innervation in the limbic system. Brain 128, 1314–1322. 10.1093/brain/awh445 15716302

[B65] Rodriguez-Oroz M. C. Jahanshahi M. Krack P. Litvan I. Macias R. Bezard E.. (2009). Initial clinical manifestations of Parkinson’s disease: features and pathophysiological mechanisms. Lancet Neurol. 8, 1128–1139. 10.1016/S1474-4422(09)70293-5 19909911

[B66] Rossi A. Berger K. Chen H. Leslie D. Mailman R. B. Huang X. (2018). Projection of the prevalence of Parkinson’s disease in the coming decades: revisited. Mov. Disord. 33, 156–159. 10.1002/mds.27063 28590580 PMC5720940

[B67] Samii A. Nutt J. G. Ransom B. R. (2004). Parkinson’s disease. Lancet 363, 1783–1793. 10.1016/S0140-6736(04)16305-8 15172778

[B68] Santamaria-Garcia H. Baez S. Reyes P. Santamaria-Garcia J. A. Santacruz-Escudero J. M. Matallana D.. (2017). A lesion model of envy and Schadenfreude: legal, deservingness and moral dimensions as revealed by neurodegeneration. Brain 140, 3357–3377. 10.1093/brain/awx269 29112719 PMC5841144

[B69] Schott B. H. Niehaus L. Wittmann B. C. Schutze H. Seidenbecher C. I. Heinze H. J.. (2007). Ageing and early-stage Parkinson’s disease affect separable neural mechanisms of mesolimbic reward processing. Brain 130, 2412–2424. 10.1093/brain/awm147 17626038

[B70] Sprengelmeyer R. Young A. W. Mahn K. Schroeder U. Woitalla D. Büttner T.. (2003). Facial expression recognition in people with medicated and unmedicated Parkinson’s disease. Neuropsychologia 41, 1047–1057. 10.1016/s0028-3932(02)00295-6 12667540

[B71] Stevens J. P. (2002). Applied Multivariate Statistics for the Social Sciences. Mahwah, NJ: Erlbaum.

[B72] Szalisznyo K. Silverstein D. Teichmann M. Duffau H. Smits A. (2017). Cortico-striatal language pathways dynamically adjust for syntactic complexity: a computational study. Brain Lang. 164, 53–62. 10.1016/j.bandl.2016.08.005 27792887

[B73] Takahashi H. Kato M. Matsuura M. Mobbs D. Suhara T. Okubo Y. (2009). When your gain is my pain and your pain is my gain: neural correlates of envy and schadenfreude. Science 323, 937–939. 10.1126/science.1165604 19213918

[B74] Teichmann M. Rosso C. Martini J. B. Bloch I. Brugieres P. Duffau H.. (2015). A cortical-subcortical syntax pathway linking Broca’s area and the striatum. Hum. Brain Mapp. 36, 2270–2283. 10.1002/hbm.22769 25682763 PMC6869141

[B75] Tkaczynska Z. Becker S. Maetzler W. Timmers M. Van Nueten L. Sulzer P.. (2020). Executive function is related to the urinary urgency in non-demented patients with Parkinson’s disease. Front. Aging Neurosci. 12:55. 10.3389/fnagi.2020.00055 32210789 PMC7069351

[B76] Torralva T. Roca M. Gleichgerrcht E. Lopez P. Manes F. (2009). INECO Frontal Screening (IFS): a brief, sensitive and specific tool to assess executive functions in dementia. J. Int. Neuropsychol. Soc. 15, 777–786. 10.1017/S1355617709990415 19635178

[B77] Whitfield J. A. Gravelin A. C. (2019). Characterizing the distribution of silent intervals in the connected speech of individuals with Parkinson disease. J. Commun. Disord. 78, 18–32. 10.1016/j.jcomdis.2018.12.001 30612045

[B78] Zgaljardic D. J. Borod J. C. Foldi N. S. Mattis P. (2003). A review of the cognitive and behavioral sequelae of Parkinson’s disease: relationship to frontostriatal circuitry. Cogn. Behav. Neurol. 16, 193–210. 10.1097/00146965-200312000-00001 14665819

[B79] Zimmerer V. Hardy C. Eastman J. Dutta S. Varnet L. Bond R.. (2020). Automated profiling of spontaneous speech in primary progressive aphasia and behavioral-variant frontotemporal dementia: an approach based on usage-frequency. Cortex 133, 103–119. 10.1016/j.cortex.2020.08.027 33120189

[B80] Ztaou S. Lhost J. Watabe I. Torromino G. Amalric M. (2018). Striatal cholinergic interneurons regulate cognitive and affective dysfunction in partially dopamine-depleted mice. Eur. J. Neurosci. 48, 2988–3004. 10.1111/ejn.14153 30230645

