# Supplementary material for: Classifying Parkinson’s Disease Patients With Syntactic and Socio-emotional Verbal Measures
Source: Front Aging Neurosci. 2020 Nov 23;12:586233. doi: 10.3389/fnagi.2020.586233 (PMC7719774; doi:10.3389/fnagi.2020.586233)
Supplement: Supplementary file 1 [file Table_1.DOCX]

***Supplementary Material***

**Materials and methods**

**S1. General cognitive state**

The Montreal Cognitive Assessment (MoCA) (Nasreddine et al., 2005) is a sensitive cognitive screening tool for PD patients (Gill et al., 2008; Nazem et al., 2009; Dalrymple-Alford et al., 2010; Kandiah et al., 2014). It comprises 14 subtests evaluating various cognitive domains, namely: attention and concentration, executive functions, memory, language, visuoconstructional and visuospatial skills, conceptual thinking, calculations and orientation. Its highest possible score is 30.

The INECO Frontal Screening (IFS) battery (Torralva et al., 2009) is a sensitive tool for detecting executive dysfunction in neurological and neuropsychiatric disorders, including PD Its maximum possible score is 30 points.

**S2. Participants**

No significant differences were found between PD patients and controls in either the MoCA (F(1,53) = 3.41, p = .562, d = .16), the ACE-R (F(1,53) = .97, p = .329, d = .27) or the IFS total score (F(1,53) = 2.92, p = .093, d = .47). Similarly, there were no differences among PD-on and PD-off patients in the MoCA (F(1,29) = .00, p = 1.000, d = 0), the ACE-R (F(1,29) = 2.56, p = .121, d = .58), or the IFS total score (F(1,29) = 1.00, p = .325, d = .36).

**S3. Socio-emotional language task**

Participants were asked to read 15 sentences describing fortunate events and 15 sentences describing unfortunate events (see below) that involving either of the two target characters. After reading the fortunate events, participants rated how much envy they felt for the character on a 9-point Likert Scale (1 = no envy, 9 = extreme envy). After reading the unfortunate events, participants rated how much pleasure they felt (1 = no pleasure, 9 = extreme pleasure). The task also included ten neutral events used as a control condition.

**Stimuli**

*Fortunate events: envy block*

1. She/he got a good grade on the exam although she/he did not study.
2. She/he achieved sports recognition although she/he did not train.
3. She/he got a raise because she/he is friends with the boss.
4. She/he won the lottery but is the son of a multi-million-dollar family.
5. She/he managed to get accepted at the University because she/he is the son/daughter of the Dean.
6. She/he avoided cueing for two hours at the bank by simulating a disability.
7. She/he claimed the work of an employee as his own and received a promotion.
8. She/he won the race after pushing her/his rival.
9. She/he got the best student award although she/he cheated in the exams.
10. She/he received government grants even though she/he has a high salary.
11. She/he went on vacation with the money she/he saved by evading taxes.
12. She/he got a meritorious grade despite plagiarizing the thesis.
13. She/he avoided a fine by bribing the police officer.
14. She/he bought a luxury cell phone with the money he stole from her/his brother.
15. She/he got big life insurance after poisoning her husband.

*Unfortunate events:* Schadenfreude *block*

1. She/he smeared the suit with wine while mocking a woman/man for being fat.
2. She/he came drunk to the exam and failed.
3. She/he presumed to be the best speaker and failed at the conference.
4. She/he was punished at work for being late every day.
5. She/he was excluded from her/his group of friends after discovering that she/he told lies.
6. She/he hit the dog with a stick and the dog bit her/him
7. She/he pretended to have success with the opposite sex until her/his partner discovered her/him being unfaithful.
8. She/he cheated on a test and she/he was expelled.
9. She/he tried to get on a bus without paying and the other passengers accused her/him with authorities.
10. She/he fell down trying to skip the metro register.
11. She/he was discovered as being corrupt and he/she was denounced.
12. She/he was fined for driving under the influence.
13. She/he tried to cheat an older person and the police discovered her/him.
14. She/he crashed her/his car after crossing a red light.
15. She/he was penalized and had her/his Facebook account closed after posting inappropriate photos.

*Neutral events*

1. She/he bought a bag to store the clothes.
2. She/he read the latest news in her/his favorite newspaper.
3. She/he cleaned the bathroom and the kitchen of the house.
4. She/he turned off the light and closed the door before leaving home.
5. She/he took a shower in her/his house before going to work took.
6. She/he mowed the lawn of his yard last month.
7. She/he turned on the light to search for her/his glasses.
8. She/he searched for an Internet address and wrote it down in his notebook.
9. She/he locked the door before going to bed.
10. She/he brushed her/his teeth after eating.

**Supplementary Table 1. Outcomes on the syntactic processing and socio-emotional tasks**

|  | PD  patients | Controls | PD-on | PD-off | PD  vs. controls | PD  vs. controls | PD-on vs.  PD-off | PD-on vs.  PD-off |
| --- | --- | --- | --- | --- | --- | --- | --- | --- |
|  | (*n* = 31)  Mean (*SD*) | (*n* = 24)  Mean (*SD*) | (*n* = 15)  Mean (*SD*) | (*n* = 16)  Mean (*SD*) | *p*-value | *d* | *p*-value | *d* |
| Touching A with B^a^ | 9.45 (1.88) | 10.96 (1.40) | 10.07 (1.44) | 8.89 (2.09) | .002** | .91 | .07 | .66 |
| Embedded sentences ^a^ | 9.48 (.96) | 9.75 (.53) | 9.47 (1.25) | 9.50 (.63) | .22 | .35 | .92 | .03 |
| *Schadenfreude* ratings^a^ | 5.18 (1.56) | 6.51 (1.50) | 5.71 (1.10) | 4.69 (1.78) | .002** | .87 | .06 | .69 |
| Envy ratings ^a^ | 6.93 (1.22) | 6.63 (1.65) | 6.92 (1.12) | 6.94 (1.35) | .43 | .21 | .95 | .02 |
| Neutral ratings ^a^ | 3.88 (1.26) | 4.0 (.88) | 3.85 (1.28) | 3.90 (1.29) | .67 | .12 | .91 | .04 |
| PD: Parkinson’s disease; PD-on: Parkinson’s disease patients in the “on” phase of medication; PD-off: Parkinson’s disease patients in the “off” phase of medication.  ^a^ *p* values were calculated through one-way ANOVA.  **Alpha level set at .05. | | | | | | | | |

**Supplementary Table 2. Standardized coefficients of discriminant functions**

| Predictor variables | PD patients vs. controls | PD-on vs. PD-off |
| --- | --- | --- |
|  |  |  |
| Touching A with B total score | 1.000 | 1.000 |
| *Schadenfreude* ratings | 1.000 | 1.000 |
| Both domains |  |  |
| Touching A with B total score | .773 | .793 |
| *Schadenfreude* ratings | .755 | .813 |

**Supplementary references**

Dalrymple-Alford, J.C., MacAskill, M.R., Nakas, C.T., Livingston, L., Graham, C., Crucian, G.P., et al. (2010). The MoCA: well-suited screen for cognitive impairment in Parkinson disease. *Neurology* 75(19)**,** 1717-1725. doi: 10.1212/WNL.0b013e3181fc29c9.

Gill, D.J., Freshman, A., Blender, J.A., and Ravina, B. (2008). The Montreal cognitive assessment as a screening tool for cognitive impairment in Parkinson's disease. *Mov Disord* 23(7)**,** 1043-1046. doi: 10.1002/mds.22017.

Kandiah, N., Zhang, A., Cenina, A.R., Au, W.L., Nadkarni, N., and Tan, L.C. (2014). Montreal Cognitive Assessment for the screening and prediction of cognitive decline in early Parkinson's disease. *Parkinsonism Relat Disord* 20(11)**,** 1145-1148. doi: 10.1016/j.parkreldis.2014.08.002.

Nasreddine, Z.S., Phillips, N.A., Bedirian, V., Charbonneau, S., Whitehead, V., Collin, I., et al. (2005). The Montreal Cognitive Assessment, MoCA: a brief screening tool for mild cognitive impairment. *J Am Geriatr Soc* 53(4)**,** 695-699. doi: 10.1111/j.1532-5415.2005.53221.x.

Nazem, S., Siderowf, A.D., Duda, J.E., Have, T.T., Colcher, A., Horn, S.S., et al. (2009). Montreal cognitive assessment performance in patients with Parkinson's disease with "normal" global cognition according to mini-mental state examination score. *J Am Geriatr Soc* 57(2)**,** 304-308. doi: 10.1111/j.1532-5415.2008.02096.x.

Torralva, T., Roca, M., Gleichgerrcht, E., Lopez, P., and Manes, F. (2009). INECO Frontal Screening (IFS): a brief, sensitive, and specific tool to assess executive functions in dementia. *J Int Neuropsychol Soc* 15(5)**,** 777-786. doi: 10.1017/S1355617709990415.
